# Level of Evidence of Dental Research in Saudi Arabia (2000–2020)

**DOI:** 10.1155/2021/3463434

**Published:** 2021-08-17

**Authors:** Mona Rajeh, Waad Khayat

**Affiliations:** ^1^Dental Public Health Division, Preventative Dentistry Department, College of Dentistry, Umm Al-Qura University, Makkah, Saudi Arabia; ^2^Operative Dentistry Division, Department of Restorative Dentistry, College of Dentistry, Umm Al-Qura University, Makkah, Saudi Arabia

## Abstract

**Objectives:**

The level of evidence (LOE) of Saudi dental research from 2000 to 2020 was evaluated, and factors associated with the LOE were determined.

**Methods:**

This study was a systematic review. PubMed, Web of Science, and Medline databases were utilized to retrieve available dental articles published in English between January 2000 and May 2020. The inclusion criteria consisted of clinical studies conducted in Saudi Arabia with at least one Saudi dental affiliation. The retrieved eligible articles were evaluated independently by two reviewers using a modified Oxford LOE scale. The LOE of the studies was compared between the last two decades.

**Results:**

Of the 7237 articles identified, 1557 articles met the inclusion criteria. Approximately 78% of the published articles reported Level IV evidence. A higher trend toward Level I, II, and III publications has occurred in recent years (i.e., 2010–2020). However, no statistically significant difference existed in LOE proportions between the two decades. The presence of international collaboration and high journals' impact factor was significantly associated with a higher LOE.

**Conclusion:**

Most published dental research studies were low LOE studies (i.e., Level IV). National and international collaboration is highly encouraged as this is a factor, according to our findings, that would be a positive addition toward publishing dental research of a higher LOE in Saudi Arabia.

## 1. Introduction

In the early 1990s, evidence-based dentistry (EBD) was introduced and became a fundamental part of dental practice [1]. The primary aim of EBD is to improve the quality of oral health care by integrating dentists' experience, the values of the patients, and the best accessible scientific evidence [2]. Therefore, dentists are expected to regularly be updated with new techniques and procedures to provide the best treatment for their patients. In essence, EBD created a need to ensure that dentists can refer to high-evidence, valid, and reliable evidence that will guide them in their clinical decision-making in dental practice.

The level of evidence (LOE) is a tool that guides clinicians to search the clinical evidence on certain topics rather than judging the quality of evidence [3]. Based on the level of evidence, the research can be ranked from the highest evidence (Level I, i.e., randomized controlled trials) to the lowest evidence (Level IV, i.e., case reports). Generally, the results from the high level of evidence studies are more reliable and reproducible in clinical practices [4, 5]. David Sackett first introduced a ranking system to assess the methodological quality of clinical studies regarding the level of evidence. The Oxford Centre for Evidence-Based Medicine is widely used as the standard guide to rank evidence in medical and dental fields [6]. The Oxford LOE has been modified and adopted by many researchers [7–9]. Unlike other LOE assessment tools, Oxford LOE considers both the design and the outcomes of the studies.

National and international researchers conducted bibliometric studies to appraise and assess the quality of dental research [10–13]. For example, many studies have evaluated authorship, the presence of collaboration, and journal impact factors. Ul Haq et al. [14] conducted a bibliometric analysis of Saudi dentists' published research and analyzed their citation impact [14]. The authors concluded that Saudi researchers need to find more innovative research ideas to obtain global attention and more citations. However, to the best of our knowledge, no previous study has explored the level of evidence of published research in dentistry in Saudi Arabia.

Conducting high-evidence research has challenges; however, the government's directions are allocated to provide financial and the best available resources to support research. The Saudi government has prioritized health, education, and research in the *Kingdom's Vision 2030*, declared in April 2016 [15]. Hence, conducting research with a high level of evidence is possible. As the number of studies published by Saudi authors has dramatically increased [14], this study, therefore, aimed to evaluate the level of evidence of published Saudi dental research over the past 20 years and the changes in the level of evidence over time. We also investigated whether the level of evidence of Saudi dental research was affected by journal type, impact factor, number of citations, and the presence of international collaboration.

## 2. Materials and Methods

### 2.1. Search Strategy

An online search was conducted between June 2020 and December 2020 at Umm al Qura University in Saudi Arabia. The authors accessed PubMed, Web of Science, and Medline databases to search the terms “Saudi Arabia” AND “dentistry oral surgery and medicine” OR “dentistry” OR “oral surgery”. After retrieving the available studies, each article was identified by title and abstract screening. The inclusion criteria were applied (described in Section 2.2), followed by accessing the full text to extract more data.

### 2.2. Study Selection

**Figure 1:**
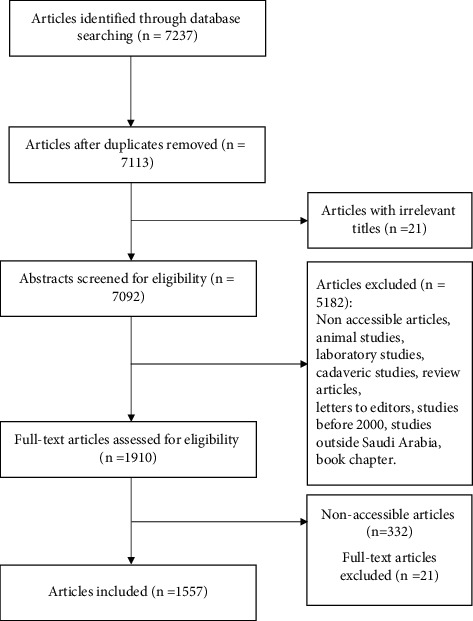
Schematic presentation of the review process.

This study included all clinical-based dental, oral, and maxillofacial studies having at least one author with Saudi dental affiliation (affiliated address in Saudi Arabia). We included studies conducted in Saudi Arabia that were published in English in any medical, dental, public health, or health science journal between January 2000 and May 2020. The authors excluded benchwork studies, animal and cadaveric studies, narrative reviews, qualitative studies, expert's opinion, letters to the editors, editorials, comments, book chapters, studies that recruited population only from outside Saudi Arabia, and studies that had no access to their full text (Figure 1). The two reviewers independently graded the studies by using the criteria of a modified Oxford Centre for Evidence-Based Medicine [16]. The articles were ranked from Level I (e.g., randomized controlled trials) to Level IV (e.g., case reports), based on the modified Oxford LOE criteria (Table 1). Level V studies (e.g., animal studies) were excluded from the review process, consistent with LOE assessments used in other medical specialties [17]. The reviewers independently conducted the screening procedures and classified the articles independently. Any disagreement was resolved by discussion until a consensus was reached.

### 2.3. Data Extraction

The following information was extracted from each article: name of the journal, journal type, impact factor, publication year, citations, study design, and whether the study was part of an international collaboration (i.e., authors having international affiliation). All data were collected in Excel (Microsoft, Redmond, WA, USA).

### 2.4. Statistical Analysis

Stata 23 (StataCorp, LLC., College Station, TX, USA) was used to conduct the statistical analysis. Descriptive statistics such as frequency and percentage were calculated. The chi-square test was used to compare between categorical variables. Statistical significance was set at *p* < 0.05. The *kappa* value was calculated to assess the level of agreement between the two reviewers. Kappa values were assessed using the criteria described by Landis and Koch [18].

## 3. Results

Of the 7237 articles identified, 1557 articles met the inclusion criteria and were included in the review process (Figure 1). Among the various study designs, cross-sectional studies were the most commonly published studies and accounted for 64.55% of the total published articles (Table 2). Systematic reviews and randomized controlled trials were the second most common published articles (20% collectively). The level of agreement between the two reviewers was almost perfect, *kappa* = 0.96 (*p* < 0.001).  Table 3 describes the benchmark scale proposed by Landis and Koch (1977).

**Figure 2:**
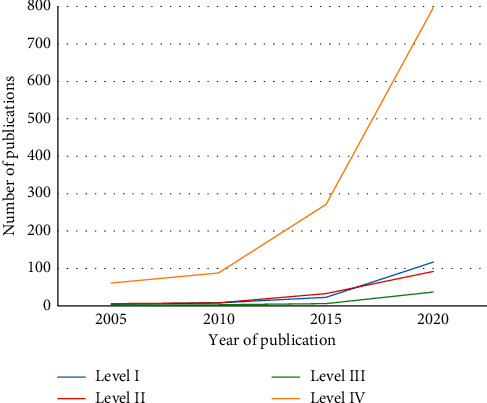
Saudi dental publication pattern based on a modified Oxford level of evidence scale.

**Figure 3:**
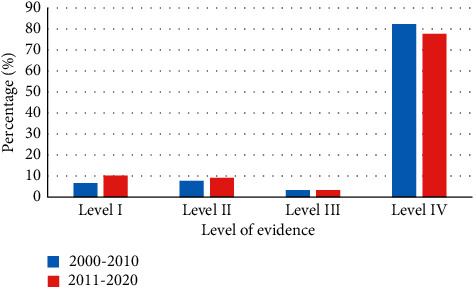
The percentage of Saudi dental published studies ranked by Oxford level of evidence scale in the last two decades.

Level IV studies constituted 78% of all publications, followed by Level I studies (10%), Level II studies (9%), and Level III studies (3%). Level IV articles were the most frequently published papers over the past 20 years. A higher trend in Level I, II, and III publications occurred in recent years (Figure 2). One hundred eighty-one studies were published between January 2000 and December 2010, and 1376 studies were published between January 2011 and May 2020. No statistically significant difference in LOE proportions existed between the 2 decades (Table 4 and Figure 3).

International collaborations with centers outside of Saudi Arabia were reported in 35% of the articles. Studies that had an international collaboration with other countries had statistically significantly higher proportions of Levels I, II, and III studies and a low proportion of Level IV studies, compared to studies without an international collaboration (*p* ≤ 0.001).

The 1557 articles were published in 306 different journals (Table 5). Approximately, 67% were published in dental journals and 33% were published in nondental journals. No significant association existed between journal type and LOE (*p* = 0.1).

The articles were published in journals with impact factors ranging 0–8.227 (median, 1.33). The results showed a significant association between the LOE and a journal's impact factor (*p* ≤ 0.001). Journals with high impact factors had significantly higher proportions of articles with a high LOE. The citation numbers of the articles ranged 0–299 (median, 8). No statistically significant association existed between the LOE and the number of citations (*p* = 0.082).

## 4. Discussion

The primary objective of our study was to evaluate the level of evidence of dental research in Saudi Arabia using a modified Oxford LOE scale. We found that the percentage of the high-evidence research (i.e., LOEs I and II) accounted for approximately one-fifth of all articles included in our study. In contrast, Level IV studies represented more than three-quarters of the overall Saudi dental publications during the past 20 years. This finding is not limited to the LOE of Saudi dental literature as most Saudi medical publications consisted of Level IV studies in different specialties in the medical field [19]. Compared to dental literature in other countries, Iranian and Brazilian dental literature studies show similar findings: Level IV evidence represented the highest prevalence of research among all other levels [11, 20–22]. In addition, a study published by Natto et al. [10], which analyzed dental articles of 33 ISI journals over the last 50 years, showed that case report and case series (i.e., Level IV) constituted the highest prevalence among all other study designs in the dental literature worldwide [10].

In general, high LOE studies such as randomized controlled trials are not always feasible to conduct because of certain ethical, financial, or other challenging factors. However, study designs with a lower LOE such as cross-sectional studies and case reports/series are much easier, faster, and less expensive to conduct. This factor explains why researchers tend to conduct more studies using these lower LOE study designs. In addition, in many clinical situations, observational study designs or clinical trials may be the most convenient methods to answer certain types of research questions, although they are generally graded with lower evidence than the controlled and randomized interventional designs. Some research methods provide better evidence than others because not all study designs are equal regarding the risk of errors and bias. Therefore, methodologies that present less bias should always be considered when applicable because they contribute to a higher evidence level while considering the adequacy of the research design in answering the research question [23].

Our study also gave insight regarding the changes in the LOE of Saudi dental research over the past two decades. During the past 10 years, the number of published Saudi dental articles has increased approximately seven times more than in the preceding decade. This remarkable growth in the number of publications is related to the increase in the number of dentists and dental institutions and graduate programs that encourage research in Saudi Arabia. With regard to dental research evidence, the percentage of Saudi high LOE dental research (i.e., LOEs I and II) has increased by 5%, whereas the percentage of lower LOE publications (i.e., LOEs III and IV) has reduced by 5% during the period from 2011 to 2020, compared to the period from 2000 to 2010. This desirable change is minor and statistically not significant, although it reflects the awareness of dental researchers to conduct more studies with higher LOE. This improvement in the LOE of Saudi-affiliated dental research over time parallels the positive changes in the LOE of dental publications in dental journals worldwide [10].

Collaboration with international dental institutions and the impact factor (IF) of a journal in which an article is published are potential indicators of the LOE. Our study found a statistically significant association between the LOE and these factors. Approximately, more than one-third of the total Saudi-affiliated dental studies had an international collaboration. The percentage of the high LOE (i.e., LOEs I and II) collaborated studies (27.8%) was approximately twice that of local studies (13.7%) with the same LOE, whereas the percentage of low LOE collaborated studies (LOEs III and IV; 72.2%) was 14.2% lower than the percentage of studies without any international collaboration (86.3%). Our finding was consistent with those of medical studies that showed a statistically significant association between international collaboration and high LOE published medical articles [5, 7, 17, 24]. Hence, international collaboration, which expands the border of experience interchanges between dental institutions and improves the LOE of research, is highly encouraged and should be facilitated.

The journal impact factor is commonly used to rank a journal based on the number of citations, to facilitate the comparison between various scientific journals in terms of quality and reputation. It is obtained by calculating the average number of citations of an article published in that journal during the two preceding years [25]. The higher the number of article citations, the greater the IF of the journal. Our study found a statistically significant positive association between the LOE and a journal's IF. A medical study by Amiri et al. (2013) had similar findings, whereas other researchers did not find any statistically significant association between a journal's IF and the LOE [4, 7, 24].

A debate exists regarding whether the IF should be viewed as a quality measure of a journal because the citation rate of published articles varies and therefore it is not representative of an article's quality [7]. Impact factors are used to assess the overall merits of a journal, but they are inadequate for evaluating the quality of an article because they are not directly related to the LOE of the articles. However, editors of journals with higher IFs are stricter about the LOE of articles they publish. Many editors have statistical advisors to ensure the quality of the articles published. Some journals may request the authors to submit the LOE rating of their clinical study, which indicates that they consider the quality of the publication [7]. No other system is as widely accepted as the IF for assessing a journal's quality [26]. Therefore, the IF is not a direct measure of the quality of an article but it can be an indicator of the LOE of publications in scientific journals because the quality of the articles and the quality of journals may influence each other.

Citation counting is one way to measure the impact of the article. However, some concerns exist when using the number of citations to evaluate the LOE of a study [13], which include the fact that recent articles have the disadvantage of receiving fewer citations than older articles. Also, articles published in open access journals may receive higher citations due to their availability and easy accessibility. In addition, published articles in less popular research topics receive fewer citations, despite the LOE of the published study. Bias may also occur as a result of citing already highly cited articles [13].

Neither the category of the journal (i.e., dental vs. nondental) nor the citation count had any statistically significant association with the LOE, as indicated by the results of our study and a study by Jamjoom et al. [24].

Our study searched three of the most commonly used databases in dental research. Each author independently accessed each article with a high interrater agreement. Among the articles found by searching these databases, 332 articles were excluded during assessing the eligibility for inclusion because they were nonaccessible, which may affect the results. Also, some journals that published Saudi-affiliated dental studies may not be indexed in these databases, and therefore they could have been overlooked.

The Oxford level of evidence (LOE) classification system is widely used in the medical literature to simplify the process of determining the strength of the evidence generated by various study designs [23]. However, the Oxford LOE scale was developed, to a large extent, to evaluate study designs that answer primarily the clinical research questions related to therapeutic interventions [27, 28]. Research questions that are related to prognostic, diagnostic, and causation studies are best answered by observational study designs; however, not all research designs are included in the Oxford LOE classification. Therefore, many previous studies have applied modifications to this classification by integrating other systems such as the Grading of Recommendations, Assessment, Development, and Evaluation (GRADE) [20, 29]. Observational studies constitute a substantial portion of dental research. Therefore, in our study, we also modified the Oxford LOE scale to evaluate various studies that provide valuable data in Saudi dental literature.

One of the limitations of our study was its focus on clinical research only. The preclinical studies, including in vitro and animal studies, which also provide vital information before designing clinical studies, were excluded when using the modified Oxford LOE system. Such preclinical and experimental study designs represented a significant part of dental research, particularly in the field of dental materials, dental tissue regeneration, and oral biology. Therefore, it is recommended for future studies to use a LOE classification scale that can evaluate the dental laboratory and animal studies as well.

Also, the current classification system used for grading the LOE of research gives a general evaluation of the clinical research, but critical appraisal of individual articles is always recommended to precisely determine the quality of a study because not all studies of the same design have the same quality. Thus, some well-designed observational studies can provide better evidence than poorly conducted RCT studies which generally provide better evidence.

Unfortunately, the dental literature has a general lack of well-designed studies due to errors with control groups, bias, randomization, blinding allocation, sample size calculations, statistical analysis, and results interpretation [10]. Therefore, the study design and ensuring the proper study structure should be considered in the evaluation of the LOE. Hence, some LOE classifications systems such as the GRADE system rate up the observational studies that have a dramatic effect for more accurate assessment and better application of the LOE [29].

This current study spots light on the level of evidence of current dental research. The LOE by itself is not an indicator of the clinical significance of the study but it is a very useful tool to categorize articles based on evidence level for evidence-based practice and for developing policies for research management and funding. We recommend future studies to objectively assess the reasons for conducting low LOE studies in dental publications in Saudi Arabia and consequently guide dental researchers to design and conduct better evidence studies in future research.

## 5. Conclusion

Based on our study's modified Oxford LOE scale, most clinical dental research in the past 20 years in Saudi Arabia had Level IV evidence. Level I was the second most common LOE, based on our study's findings, whereas Level III was the least common LOE. The presence of international collaboration and journal IF had a statistically significant association with the LOE of Saudi dental research. However, neither the category of the journal nor the citation count seems to be associated with the LOE of dental publications. The increase in the number of dental publications in the past decade has been substantial with a slight improvement of the LOE. However, we encourage Saudi researchers to conduct more studies of higher LOE to promote better EBD in Saudi Arabia.

## Figures and Tables

**Table 1:** The modified Oxford's level of evidence scale.

Level	Type of study
I	Meta-analysis, systematic reviews of RCTs, randomized controlled clinical trials (RCTs)
II	Systematic review of cohort studies, systematic reviews of clinical trials, individual cohort studies, individual clinical trials, quasiexperimental
III	Systematic review of case-control studies, individual case-control studies
IV	Systematic reviews of mixed low evidence level studies. Case series, case reports, and cross-sectional studies
V	Expert opinions without explicit critical appraisal, experimental research, animal studies, reviews

**Table 2:** Classification of the extracted articles according to the design of the study.

Study design	Frequency	(%)
Meta-analysis	5	0.32
Systematic reviews	200	12.85
Randomized controlled trials	103	6.62
Clinical trials	75	4.81
Cohort studies	16	1.02
Case-control studies	24	1.54
Cross-sectional studies	1005	64.55
Case reports	119	7.64
Case series	10	0.64
Total	1557	100

**Table 3:** Interpretation of kappa values by Landis and Koch scale.

Kappa statistics	Strength of agreement
<0.00	Poor
0.00–0.20	Slight
0.21–0.40	Fair
0.41–0.60	Moderate
0.61–0.80	Substantial
0.81–1.00	Almost perfect

**Table 4:** Level of evidence of dental research in Saudi Arabia.

Feature	Article numbers	Level of evidence *n* (%)				*p* value (significance)
		I	II	III	IV	
*Year*						
2000–2010	181	12 (6.63)	14 (7.73)	6 (3.31)	149 (82.32)	0.414 (NS)
2011–2020	1376	140 (10.17)	125 (9.08)	43 (3.12)	1068 (77.62)	

*International collaboration*						
Yes	554	88 (15.88)	66 (11.91)	32 (5.78)	368 (66.43)	*p* ≤ 0.001 (Sig)
No	1003	64 (6.38)	73 (7.28)	17 (1.69)	849 (84.65)	

*Journal*						
Dental	1040	104 (10)	104 (10)	36 (3.46)	796 (76.54)	0.1 (NS)
Others	517	48 (9.28)	35 (6.77)	13 (2.51)	421 (81.43)	

*Journal*						
Saudi	278	33 (11.87)	25 (8.99)	11 (3.96)	209 (75.18)	0.452 (NS)
Non-Saudi	1279	119 (9.3)	114 (8.91)	38 (2.97)	1008 (78.81)	

*Journal's IF*						
IF > 1	988	115 (11.64)	106 (10.73)	40 (4.05)	727 (73.58)	*p* ≤ 0.001
IF < 1	569	37 (6.5)	33 (5.8)	9 (1.58)	490 (86.12)	(Sig)

*Citation numbers*						
Citations >10	693	77 (11.11)	80 (11.54)	24 (3.46)	512 (73.88)	0.082 (NS)
Citations <10	864	75 (8.68)	59 (6.83)	25 (2.89)	705 (81.6)	

IF: impact factor; Sig: significant; NS: not significant.

**Table 5:** Frequencies of Saudi dental publications among different journals.

Journal type	Frequency	(%)
Dental	1034	66.41
Medical	386	24.79
Health Science and Technology	69	4.43
Science	53	3.4
Pharmacology	15	0.96

## Data Availability

The data that support the findings of this study are available on request from the corresponding author.

## Abbreviations

EBD: Evidence-based dentistry
IF: Impact factor
LOE: Level of evidence
RCT: Randomized controlled clinical trial
GRADE: Grading of Recommendations, Assessment, Development, and Evaluation.
